# Ethnobotanical study of medicinal plants in Adwa District, Central Zone of Tigray Regional State, Northern Ethiopia

**DOI:** 10.1186/s13002-021-00498-1

**Published:** 2021-12-24

**Authors:** Muhidin Tahir, Letebrhan Gebremichael, Tadesse Beyene, Patrick Van Damme

**Affiliations:** 1 Department of Biology, College of Natural and Computational Sciences, Oda Bultum University, P.O. Box 226, Chiro, Ethiopia; 2 Abbiyi-Addi College of Teacher and Educational Leadership, P.O. Box 11, Mekelle, Ethiopia; 3 Department of Biology, College of Natural and Computational Sciences, Mekelle University, P.O. Box 231, Mekelle, Ethiopia; 4 Laboratory for Tropical and Subtropical Agriculture and Ethnobotany, Department of Plants and Crops, Faculty of Bio-Science Engineering, Ghent University, Coupure links 653, 9000 Gent, Belgium; 5 Faculty of Tropical AgriSciences, Czech University of Life Sciences Prague, Kamycka 129, 165 21 Prague 6, Suchdol Czech Republic

**Keywords:** Ailments treated, Indigenous knowledge, Medicinal plants, Adwa District, Ethiopia

## Abstract

**Background:**

Medicinal plants have been used for ages in Ethiopia. Some 887 plant species have been documented to heal human and livestock health problems. Documenting the traditional use of medicinal plants is a vital step in obtaining information on bioactive chemicals, preserving indigenous knowledge and ultimately interesting, medicinal plant species. We conducted this study with the aim of documenting the ethnobotanical knowledge associated with medicinal plant use in Adwa District, Northern Ethiopia.

**Methods:**

The study was conducted from September 2018 to December 2019. A total of 393 informants (242 males and 151 females) were selected. Data were collected using semi-structured interviews, guided walks and group discussions. We calculated informant consensus factors (ICF) and fidelity level (FL) and performed ranking and paired comparisons. Data were analysed using descriptive statistics, including independent sample *t* test and ANOVA.

**Results:**

Overall, we documented 127 medicinal plants belonging to 105 genera, under 54 families to be used by local people to address 43 human and 15 livestock ailments. Families Fabaceae and Solanaceae were the most important ones with 11 (8.66%) species each. Herbs were the dominant growth form (55 species), followed by shrubs (39). The most frequently used plant parts were leaves (24.27%) followed by roots (14%). The most important preparation method was crushing and pounding (42.7%) followed by fumigation (smoke and vapour) (23, 11.1%). The common route of administration was via skin application (67 or 32%) followed by oral (63, 27%). ICF showed that tonsillitis had the higher value (0.95). *Cucumis ficifolius* A. Rich. was the most preferred medicinal plant (36) treating abdominal pain, followed by *Kalanchoe quartiniana* A. Rich. for blackleg (34)*.*

**Conclusions:**

Adwa District is relatively rich in medicinal plant diversity and traditional knowledge on use, preparation and application of the medicinal flora. However, agricultural expansion (51%), overgrazing (43%) and drought (37%) were mentioned most when informants were asked about the threats to medicinal plants in Adwa District. Hence, on-site and off-site medicinal plant conservation would help protect medicinal plants in the district.

**Supplementary Information:**

The online version contains supplementary material available at 10.1186/s13002-021-00498-1.

## Introduction

Plants have been used in traditional medicine since well before the advent of the so-called modern or allopathic medicine to address a wide range of different health problems [1–5]. Documents show that in China plants were already used in traditional remedies 7000–6000 years ago [6]. Other records show similar uses by Egyptians, Hebrews, Babylonians and Syrians dating back to 1600 BC [7]. Any local, specific medicinal plant use is the result of a combination of different cultural backgrounds, beliefs and attitudes that all refer to specific plant resources [8, 9]. The indigenous knowledge on plants used in traditional medicine is an important factor that helps to define cultural identities of communities and provide evidence for links to their past, and land and plant use practices [10]. Studying traditional knowledge on medicinal plant is also a necessary basis for finding new lead compounds for developing new modern, allopathic drugs [11].

Nearly 85% of the world population uses medicinal plants to alleviate different human and animal diseases [2, 12]. Besides, for nearly 80% of people in developing nations this is the only medicinal resource available to them for lack of money or physical access to allopathic drugs [13]. The majority of the populations (80%) in Africa primarily relies on traditional medicinal plants for their primary health care system [1]. In addition to the expensiveness of modern therapies, the high spiritual and cultural acceptability of traditional herbal remedies is another important factor explaining the popularity of traditional medicine in developing countries [14].

In Ethiopia, the different edaphic conditions, in conjunction with a range of topographies, ranging between the high altitudes of Semien Mountain to the Afar Depression, yield a huge diversity of medicinal plant species [15, 16] especially as the going hypothesis is that extreme environmental situations bring about special coping adaptations at plant level often involving special phytochemicals. Besides, the presence of more than 70 ethnic groups results in Ethiopia having a wealth of indigenous knowledge [16, 17].

Besides, Ethiopia is also one of the eight world centres of origin and diversity of agricultural products [18]. Nearly 6500–7000 plant species among which 12% endemic species were described to be present in Ethiopia [19]. Nearly 90% of the country’s livestock and 80% of humans depend on plant-derived traditional medicine to prevent, alleviate and treat a variety of health problems [20]. Moreover, about 887 plant species have been reported to be utilized as traditional medicine in Ethiopia [21]. Widespread traditional medicinal plant use in Ethiopia can be explained by the ready acceptability of these plants from a cultural and spiritual perspective, their proven effectiveness, easy access and low financial cost in sourcing [22, 23]. Despite the great role for food and medicine of plants in Ethiopia, little has so far been done to appropriately investigate and document medicinal plants and related knowledge [24]. Besides, traditional medicinal plant use knowledge in Ethiopia is under pressure since it mostly resides with older people who are gradually disappearing, whereas there are no written records on these plants as knowledge is mainly passed orally [24, 25]. Furthermore, deforestation, overexploitation of natural resources and overgrazing are seriously threatening medicinal plants and the associated knowledge in the country [19, 26].

People in the Tigray region in general and the people of Adwa District in particular highly depend on medicinal plants because of their high acceptability from a cultural perspective, availability near their living environment, economic affordability and efficacy against certain types of human and livestock health problems. However, medicinal plants and indigenous knowledge in Adwa District are threatened by agricultural expansion, deforestation, and poor documentation and oral transfer of traditional plant knowledge. Although there is one study on the plants used in traditional medicine in our study district [27], information regarding the status of knowledge transfer, comparison of remedial plant knowledge among different social groups of the community, ranking of medicinal plants according to their curative potential, threats to the plant resource base and current conservation status of the plants was not provided. Besides, the latter study documented only very few medicinal plant species (*n* = 25). Therefore, we conducted this study with the aim of providing a more comprehensive documentation of the ethnobotanical knowledge associated with medicinal plant use by the people of Adwa District, Northern Ethiopia.

## Materials and methods

### Description of the study area

**Fig. 1:**
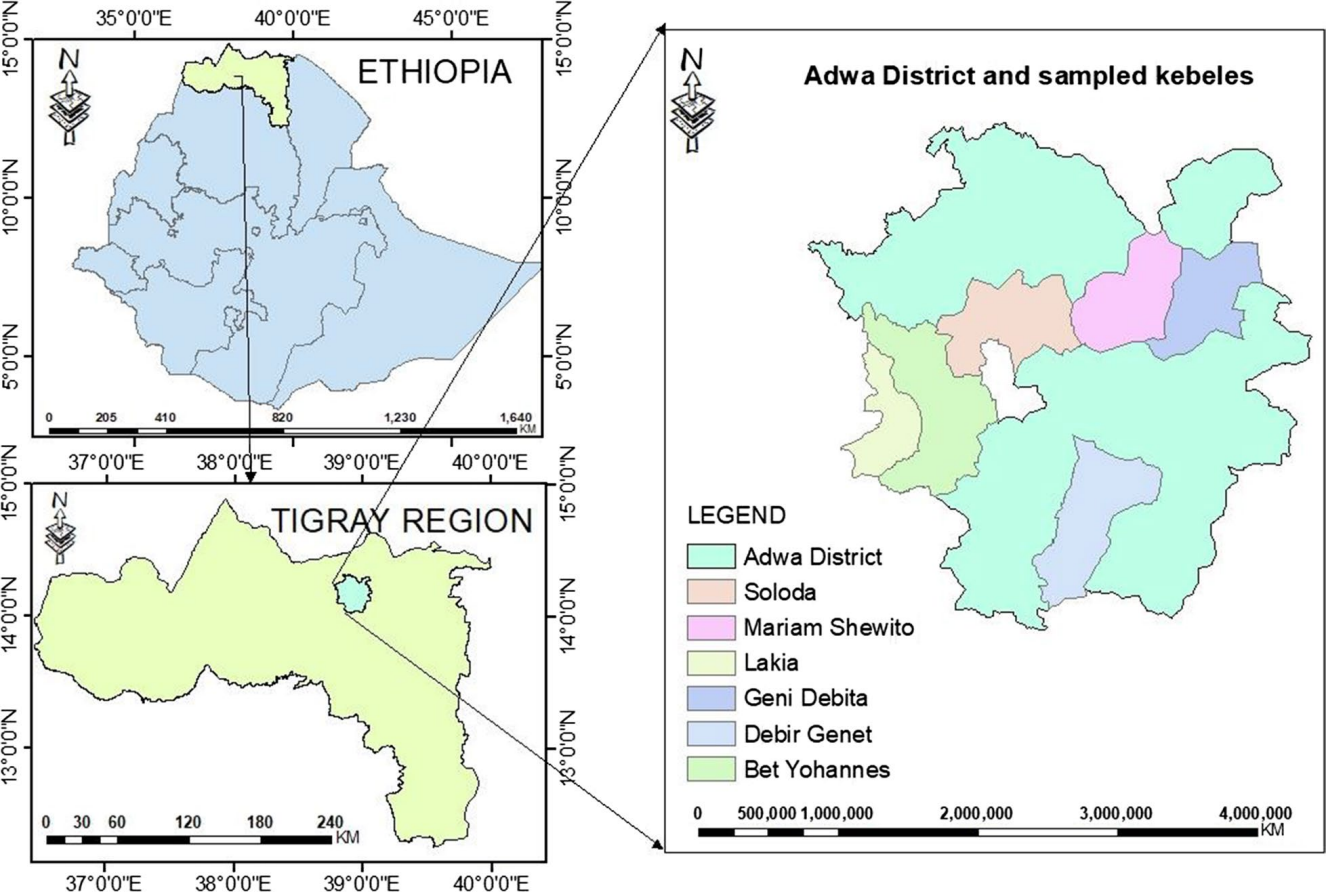
Map of Adwa District and sampled kebeles

The study was conducted in Adwa District located at 14°15′N and 38°52′E, with an elevation ranging from 1500 to 2700 m a.s.l. in Tigray Regional State of Ethiopia (Fig. 1). Tigray is one of the nine regional states of Ethiopia. It is bordered by Sudan to the West, Eritrea to the North, Afar National Regional State to the East and Amhara National Regional State to the Southwest [28]. Adwa is bounded by Werielehe to the South, La'ilaymaychew to the West, Mereblehe to the North and Enticho to the East.

An analysis of 27 years (1992–2018) of meteorological data showed that the mean annual temperature of the study area was 20 °C, with mean minimum and maximum temperatures at 7.8 °C and 30.9 °C, respectively. The hottest months were March, April and May while November, December and January were the coldest. Mean annual rainfall for the study area was 799 mm (National Meteorological Services Agency, unpublished data of 2018).

According to CSA [29], total population of the District was at 112,794, of whom 55,551 were males and 57,243 females. A total of 20,460 males-led and 8772 females-led households were counted in the District [29]. The agro-ecological zones of the study district are midland (*Weina-dega*) and lowland (*Qolla*) covering 63% and 37% of total surface, respectively (Adwa District Agricultural office, unpublished data of 2014). Crop cultivation and livestock rearing production systems are the most common agricultural activities practised in the district. Crops such as barley, wheat, pulses, *teff*, maize and sorghum are commonly cultivated in the district. Besides, vegetables and fruits are cultivated in the riverside valleys of the fertile lowlands (Adwa District Agricultural office, unpublished data of 2014). Out of a total area 65,616.705 ha in the district, 27% is protected, followed by grazing land (26.4%). Soils are mainly silt (55.82%) (*qeyih hamed*), followed by loam (*walka*) (24.34%) and clay (19.75%) (Adwa District Agricultural Office, unpublished data of 2014).

### Sample size and informant selection

Six study *kebeles* (wards; smallest administrative units), i.e. Soloda, Debre Genet, Bet-johannis, Gendebta, Lakia and Mariam Shewito (Fig. 1), were selected from a total of 18 *kebeles* in the study District based on recommendations of local elders, local authorities, presence of traditional healers, vegetation cover and agro-climatic conditions. A total of 393 informants (242 males and 151 females) were selected in different age classes. Twenty herbal healers were selected purposefully, whereas 373 general informants were selected randomly. To ensure a representative sample for the six *kebeles*, sample size was determined using Cochran’s sample size formula as presented by Bartlett et al. [30]:

$$n \, = \, N/1 + \, N{ (}e{)}^{2} ,$$

where *n* is the research sample size, *N* total number of households in all six selected *kebeles*, *e* is the maximum variability or margin of error of 5% (0.05), whereas 1 is the probability of the event occurring. As a result, we got a total sample size of:

$$n \, = \, 10,365/1 + 10,365\left( {0.05} \right)^{2} ,\;{\text{or}}\;n = \, 393.$$

The sample size of each *kebele* was determined based on the proportion of the number of households (HH) in the respective *kebeles*. For example, total household number of Bet-yohannis was 1606, yielding a number of 61 (*n* = 160 × 393/10,365 = 61). The same calculation was used for the other *kebeles* resulting in Lakia (HH = 1343, *n* = 51), Mariam Shewito (HH = 1898, *n* = 72), Gendebta (HH = 2337, *n* = 89), Soloda (HH = 992, *n* = 37) and Debre Genet (HH = 2189, *n* = 83).

### Data collection

Ethnobotanical data were collected from September 2018 to December 2019 following the methods used by [31, 32]. Semi-structured interviews were employed to obtain information on medicinal plant use, involving method(s) of preparation, routes of administration, plant parts used, threats to and conservation of these medicinal plants, based on the method adopted by Martin [31]. Interviews were undertaken based on a checklist of questions prepared in English and then translated into ‘*Tigrigna*’.

We also conducted guided field walks to make notes on local name(s), preparation, parts used, diseases treated and all other relevant information on the respective plant species. At the same time, we collected voucher specimens from the wild and in homegardens.

A brief group discussion with key informants was organized to record the status, threats and conservation status of the plants used in traditional medicine. Information obtained during the discussion was carefully recorded and analysed as indicated by Martin [31].

### Specimen collection and identification

Plant species were identified in the field and in the herbarium, using taxonomic literature, comparison with reference voucher specimens, expert assistance and using various books of the flora of Ethiopia and Eritrea [33–40]. Plant specimens (with their local name, collection number, date of collection, collector’s name, location and description of the plant) were pressed, dried, labelled and taken to the National Herbarium, Addis Ababa University for further identification.

### Data analysis

Comparison analysis of medicinal plant knowledge between different social groups was done using SPSS (version 20) software. One-way analysis of variance (ANOVA) was performed to check the significant differences of mean knowledge on medicinal plants reported by different social groups. *T* test was carried out to compare medicinal plant knowledge between gender groups (married and single) and healing experience (key and general informants). Furthermore, Microsoft Excel Spreadsheet (2010) was used to compute percentages and sums and to tabulate and draw graphs.

If different plants were prescribed for similar ailments, informants were asked to indicate their preference for a certain plant against specific diseases. Hence, preference ranking was done for the most important plants used to cure specific disease using Martin’s method [31]. Each informant attributed values to their preferred plant against certain illnesses.

We conducted paired-wise comparisons to analyse the degree of preference or level of importance of certain selected plants following [31]. Lists of pairs were presented to selected respondents. Finally, we recorded their responses and total values were summarized.

In order to evaluate the reliability of the information obtained, informants were contacted at least twice for the same topics, and correctness of information was checked and consolidated. If the second idea of the informant differed from the original information, it was rejected as irrelevant information. Only the relevant ones were retained and statistically analysed following the method adopted from Alexiades [41].

Participant agreement on plant use as traditional remedies against groups of ailments was done following [42] using the formula:

$${\text{ICF }} = {\text{ Nur}}{-}{\text{ Nt}}/{\text{Nur }}{-} \, 1,$$

where Nur is the number of use reports for a certain illness group, and Nt is the number of plants used for a specific ailment category by all informants.

To analyse the importance of plants for a given remedy, fidelity level was calculated using the following formula:

$${\text{FL }} = \, I_{{\text{p}}} /I_{{\text{u}}} \times 100,$$

where *I*_p_ is the number of respondents who independently mentioned the use of the specific plant for the same illness, and *I*_u_ is the overall number of respondents who cited the plant for any ailment in general [43].

## Results

### Socio-demographic characteristics of respondents

The informants in the study area covered different age groups, i.e. knowledgeable elders, males/females and youngsters, and education level. The majority of the informants in the study area were males (61.6%), whereas 38.4% were females. Informant age ranged from 21 to 40 years (24.9%) whereas 49.9% were between 41 and 60 years and 25.2% above 61. All informants belonged to the Orthodox religion and the Tigrian ethnic group. All informants were farmers. Most informants (172, 43.8%) were illiterate (Table 1).

**Table 1:** Medicinal plant knowledge among different social groups of the study area (*n* = 393)

Parameter	Category	*N*	Mean	*p* value
Sex	Males	242	5.98	0.972
	Females	151	5.97	
Healing experience	Key informants	20	12.95	0.0001
	General informants	373	5.6	
Age groups (in year)	21–31	40	3.5	0.0001*
	32–41	60	4.85	
	42–51	104	5.6	
	52–61	100	6.6	
	62–71	71	7.4	
	72–81	14	7.5	
	> 82	4	11	
Education level	Illiterate and elementary school	355	6.2	0.0001*
	High school and diploma	34	4.3	
	Bachelor of sciences/Bachelor of art	4	4.2	

*Significant difference at (*p* < 0.05) between averages of the paired categories

A two-tailed independent sample *t* test comparison of medicinal plant knowledge between male and female informants in the study area showed that there was no significance knowledge difference (*p* > 0.05) between them. The mean value for males was (5.98) whereas for females it was 5.97 (Table 1). Significant difference (*p* < 0.05) between key and general informants was observed in mean number of medicinal plant known and used in the study area: key informants were more knowledgeable (12.95) than general informants (5.6).

There was a significant medicinal plants knowledge difference between age groups in the study area (*p* < 0.01) (Table 1). Besides, there was also a significant medicinal plant knowledge difference (*p* < 0.05) between different education levels (Table 1).

### Medicinal plant diversity, their habitats and growth form

Overall, 127 medicinal plants belonging to 105 genera in 54 plant families, were documented to be used by the people of Adwa District. In the study district, 87 (68.5%) medicinal plants were used to alleviate human health problems only, 28 (22.05%) to treat both human and animal disease and 12 (9.45%) to cure livestock health problems only (see Additional file 1). Families Fabaceae and Solanaceae were best-represented accounting for 11 species (8.66%) each; they were followed by Asteraceae 8 (6.29%), Malvaceae, Euphorbiaceae and Lamiaceae 6 (4.72%) each, Boraginaceae 4 (3.14%) and Brassicaceae 3 (2.36%).

People in Adwa District collect medicinal plants from different sources, including from the wild, homegardens, crop field, riversides and roadsides. Out of 127 medicinal plants, 51 (40%) were gathered from natural vegetation, 34 (27%) from homegardens, 32 (25%) from crop fields and 10 (8%) from riverside.

The habits (growth forms) of remedial plants in Adwa District were herbs, shrubs, trees and climbers. The dominant growth form was herbs with 55 species (43%), followed by shrubs (39, 31%), trees (28, 22%) and climbers (5, 4%).

### Used plant parts, mode of preparation and administration

**Fig. 2:**
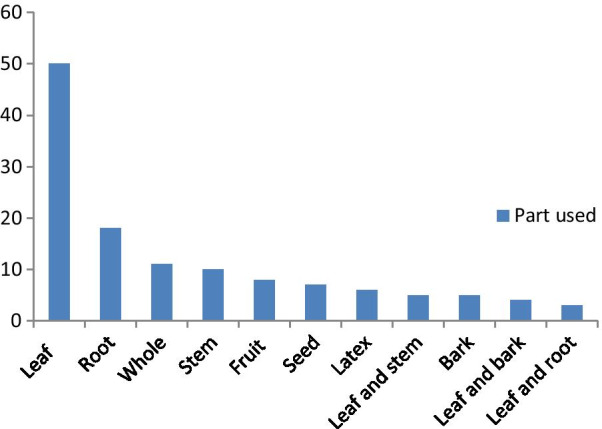
Parts of medicinal plants used by the local people in the study district

In Adwa District, various plant parts—including leaf, stems, roots, bulbs, seeds, fruits, bark, latex and whole plants—are used in traditional medicine. The most widely used plant parts were leaves which accounted (37%, 50 species) followed by roots (14%, 18) (Fig. 2).

Traditional medicines in Adwa District are prepared from fresh or dry, and both fresh and dry plant parts. The highest number, i.e. 84 (66%) of remedial plants, were prepared fresh followed by both fresh and dried (23, 18%) and dry (20, 16%).

**Fig. 3:**
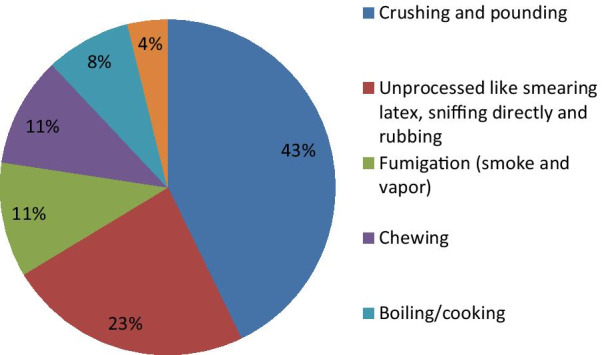
Methods used in the preparation of remedies

Local people, including the healers of the study district, agreed that traditional medicines are prepared through different methods. Most are prepared by crushing and pounding (covering 89 species; 42.7%) followed by direct consumption (without any processing; 49 species (16.3%) (Fig. 3).

The people of the study area prepare herbal medicine using various ingredients and solvents, such as coffee, tea, honey, water, milk, oil, butter, salt or water, but also consume direct. Most herbal medicines (48%) in the study area are prepared directly without any ingredients, 37% of them are diluted in water and 6% with fresh butter (see Additional file 1).

**Fig. 4:**
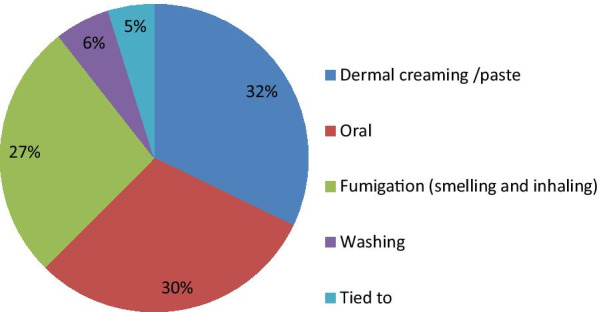
Route of administration

Traditional medicine is administered through various routes, including skin, oral, through fumigation and inhaling. The principal route of administration in the study area was through the skin (67 species or 32%) followed by oral uptake (63/30.2%) (Fig. 4).

### Human and livestock ailments treated

A total of 87 (68.5%) ethnomedicinally important plant species used to heal 43 human ailments were recorded in the six *kebeles* of Adwa District (see Additional file 1). The most common diseases which seem to affect humans in Adwa District are cough, abdominal pain, tuberculosis, malaria, tonsillitis, paralysis, diarrhoea, jaundice, amoeba and arthritis. Herbal practitioners are visited frequently for different diseases, including ‘evil eye’, febrile illness or ‘*Michi*’ and spider poison. The local communities prefer to consult traditional healers for such diseases rather than take modern medication. A number of human ailments can be healed by a single plant, whereas many medicinal plant species can cure a single disease. For instance, wounds were reported to be treated by 16 medicinal plant species, followed by cough which is treated by six medicinal plants (see Additional file 1).

In Adwa District, a total of 15 livestock ailments were recorded to be treated by 12 medicinal plants (see Additional file 1). Diseases, such as black leg (shivering), wounds around mouth and hooves, neck swelling/liver fluke, fleas and lice, acute swelling, horn worm (‘*Hasekaresi*’), cough, blotting, tuberculosis, anthrax, leech and bone fracture are common livestock health problems in Adwa District (see Additional file 1). A number of livestock ailments can be cured by a single plant, whereas a number of plants can heal a single ailment. For instance, black leg (shivering) and swelling were reported to be treated by two medicinal plants each (see Additional file 1).

We calculated the informant consensus factor to check informants agreement on the medicinal plants reported against a group of ailments. Medicinal plants used against ‘*Michi*’, febrile illness and headache had the highest ICF value (0.97), followed by tonsillitis, eye disease and ear problem disease categories (0.95) (Table 2).

**Table 2:** Informant consensus factor for six categories of ailments

Ailment category	Plant species	Use Citation	ICF	Rank
'*Michi*', febrile illness and headache	8	243	0.97	1st
Tonsillitis, bleedings and injury	8	140	0.95	2nd
Evil eye and spirit	8	65	0.89	3rd
Sprain, fire burn and toothache	5	36	0.89	3rd
Abdominal pain, parasite, ascariasis, taeniasis, tape worm and amoebiasis	20	163	0.88	4th
Wounds and traumas	8	44	0.84	5th

Fidelity level of some commonly used medicinal plants was calculated. The species, *Kalanchoe quartiniana* A. Rich. was having high fidelity level which accounted for 0.98 (98%) followed by *Rhamnus prinoides* L’Herit 0.93 (93%) (Table 3).

**Table 3:** Fidelity level of some common medicinal plants

Scientific name	Main ailment treated	IP	IU	FL	%	R
*Kalanchoe quartiniana* A. Rich	Blackleg	108	110	0.98	98	1st
*Rhamnus prinoides* L’Her	Uvula enlargement	52	56	0.93	93	2nd
*Argemone mexicana* L	Wound	27	30	0.9	90	3rd
*Withania somnifera* (L.) Dunal	Paralyses	81	91	0.89	89	4th
*Schinus molle* L	Tonsillitis	48	59	0.81	81	5th
*Clematis hirsuta* Guill. and Perr	Tonsillitis	45	57	0.79	79	6th
*Cucumis ficifolius* A. Rich	Abdominal pain	105	142	0.74	74	7th
*Aloe vera* (L.) Burm.f	Malaria	66	90	0.73	73	8th
*Solanum incanum* L	Swelling	37	51	0.73	73	8th
*Artemisia abyssinica* Sch.Bip. ex A. Rich	Evil eye	10	15	0.67	67	9th
*Plumbago zeylanica* L	Jaundice / ‘Sray’	23	37	0.62	62	10th
*Euclea racemosa* L	Injury/Fresh wound	22	37	0.59	59	11th
*Croton macrostachyus* Hochst. ex Delile	Jaundice	36	69	0.52	52	12th

Preference ranking of five medicinal plants used to alleviate human abdominal pains was done through interviewing eight key informants. The highest value (5) was assigned to plants considered to be the most effective, whereas the lowest value (1) was given to the least-effective medicinal plants. *Cucumis ficifolius* A. Rich. was thus shown to be the most preferred species (36) curing abdominal pain, followed by *Solanum incanum* L. (31) (Table 4).

**Table 4:** Preference ranking of five medicinal plants in treating abdominal pain

Medicinal plant	Key informant								Total	Rank
	k-1	k-2	k-3	k-4	k-5	k-6	k-7	k-8		
*Cucumis ficifolius* A. Rich	5	5	5	3	5	4	4	5	36	1st
*Solanum incanum* L	4	3	4	5	4	5	3	3	31	2nd
*Senna singueana* (Delile) Lock	3	4	3	4	3	2	2	4	25	3rd
*Otostegia integrifolia* Benth	2	1	1	2	1	3	5	1	16	4th
*Sida schimperiana* Hochst. ex A. Rich	1	2	2	1	2	1	1	2	12	5th

Likewise, we performed the same preference ranking on seven species used against febrile illness. *Withania somnifera* (L.) Dunal scored the highest value (49), followed by *Zehneria scabra* Sond. and *Cynoglossum lanceolatum* Forssk. (42 each) (Table 5).

**Table 5:** Preference ranking of seven medicinal plants in treating febrile illness (‘*Michi*’)

Medicinal plants	Key informant								Total	Rank
	k-1	k-2	k-3	k-4	k-5	k-6	k-7	k-8		
*Withania somnifera* (L.) Dunal	7	6	7	5	6	4	7	6	49	1st
*Zehneria scabra* Sond	5	2	6	6	7	7	5	4	42	2th
*Cynoglossum lanceolatum* Forssk	6	7	5	2	3	6	6	7	42	2nd
*Leonotis nepetifolia* (L.) R.Br	4	3	4	3	5	5	4	5	33	3rd
*Abutilon bidentatum* Hochst. A. Rich	3	4	3	7	4	3	3	3	30	4th
*Plumbago zeylanica* L	2	5	2	4	2	2	2	2	21	5th
*Vernonia amygdalina* Del	1	1	1	1	1	1	1	1	8	6th

Preference ranking for five medicinal plant species used to cure black leg in livestock showed that *Kalanchoe quartiniana* A. Rich. was the most preferred (34), followed by *Synadenium grantii* Hook.f. (29) (Table 6).

**Table 6:** Preference ranking of five medicinal plant species in treating black leg (‘*Halafien*’)

Medicinal plants	Key informants								Total	Rank
	k-1	k-2	k-3	k-4	k-5	k-6	k-7	k-8		
*Kalanchoe quartiniana* A. Rich	4	4	4	3	5	5	5	4	34	1st
*Synadenium grantii* Hook.f	5	5	5	2	1	2	4	5	29	2nd
*Rumex abyssinicus* Jacq	3	3	3	5	4	4	3	3	28	3rd
*Melia azadirachta* L	2	2	1	1	2	3	2	2	15	4th
*Rumex nervosus* Vahl	1	1	2	4	3	1	1	1	14	5th

Direct matrix ranking of seven selected multipurpose medicinal plants for six different use categories based on information gathered from eight key informants showed that *Olea europaea* L. was the most multi-used species (250), followed by *Cordia africana* Lam. (248) (Table 7).

**Table 7:** Direct matrix ranking of seven multipurpose medicinal plants

Criterion	Multipurpose plant species						
	*Olea europaea* L	*Cordia africana* Lam	*Eucalyptus globulus* Labill	*Croton macrostachyus* Hochst. ex Delile	*Ziziphus spina-christi* (Mill.) Georgi	*Senna singueana* (Delile) Lock	*Melia azadirachta* L
Medicine	20	39	40	49	24	25	27
Firewood /charcoal	55	23	39	24	40	33	10
Construction	49	45	47	32	26	12	13
Food	49	53	39	29	27	13	14
Furniture	29	55	27	13	49	36	15
Total	250	248	214	198	191	154	89
Rank	1st	2nd	3rd	4th	5th	6th	7th

Paired comparisons of five commonly used medicinal plants used to treat temporal paralysis and human limbs’ pain (eight key informants) showed that *Withania somnifera* (L.) Dunal was the most commonly used species with a score of 25, followed by *Plumbago zeylanica* L. (22) (Table 8).

**Table 8:** Paired comparison of five commonly used medicinal plants to treat paralysis and limb pain

Medicinal plant	Key informant								Total	Rank
	K-1	K-2	K-3	K-4	K-5	K-6	K-7	K-8		
*Withania somnifera* (L.) Dunal	3	2	4	4	3	1	4	4	25	1st
*Plumbago zeylanica* L	2	4	1	2	4	4	2	3	22	2nd
*Cucumis ficifolius* A. Rich	4	3	2	3	2	3	2	2	21	3rd
*Aloe vera* (L.) Burm.f	0	1	1	1	0	2	1	1	7	4th
*Croton macrostachyus* Hochst. ex Delile	1	0	2	0	1	0	1	0	5	5th

Informant consensus analysis showed that some medicinal plants are more popular than others to treat livestock ailments. Accordingly, *Kalanchoe quartiniana* A. Rich. was the most cited plant species (108), followed by *Rumex nervosus* Vahl. (Table 9).

**Table 9:** Informant consensus of eight medicinal plants used to treat blackleg ailment

Plant species	Ailment	Citation	Rank
*Kalanchoe quartiniana* A. Rich	Black leg	108	1st
*Rumex nervosus* Vahl	Black leg	32	2nd
*Rumex nepalensis* Spreng	Black leg	24	3rd
*Croton macrostachyus* Hochst. ex Delile	Black leg	23	4th
*Synadenium grantii* Hook.f	Black leg	21	5th
*Brucea antidysenterica* J.F.Mill	Black leg	10	6th
*Melia azadirachta* L	Black leg	7	7th
*Schinus molle* L	Black leg	6	8th

### Threats to medicinal plant knowledge and use

The main threat to the medicinal plant resource basis mentioned by the informants was agricultural expansion 202 mentions (51%), followed by overgrazing (168/43%), drought (145/37%), deforestation (121/31%), lack of awareness (uprooting; 108/27%), herbicide use (19/5%), invasive species (15/4%) and fire (2/0.5%).

Informant recommended more than one protection method. Accordingly, planting medicinal plants was the most recommended method to conserve and protect herbal medicines mentioned by 192 (49%) informants, followed by soil and water conservation (179/46%), protected area (159/41%) and creating awareness (115/30%).

## Discussion

The high number of men respondents in the study area might be due to the fact that traditional healers usually prefer to pass their indigenous medicinal plant knowledge to other men. Similarly, dominance of men was also reported in other studies conducted in Ethiopia [5, 16, 17, 45, 48].

A diverse set of plant species is used by the people of Adwa District (*n* = 127) for medicinal purposes. The number of medicinal plant species documented in Adwa District was higher than that from similar ethnobotanical studies in Ethiopia, i.e. in Kilte Awulaelo District 114 species were mentioned [47], in Hawassa Zuria District *n* = 105 [5], in Babile District *n* = 51 [48], Ghimbi District *n* = 49 [49], Dirre Sheikh Hussein heritage site *n* = 87 [50] and Sheko ethnic group *n* = 71 [26]. The wide utilization of medicinal plants in Adwa District might be due to insufficient provision of formal therapies, higher reliance on traditional medicine from a cultural perspective and the relative low cost of the plants used in traditional medicine.

The occurrence of a higher number of medicinal plants (*n* = 87) to treat human diseases in Adwa District might be due to the high prevalence of human ailments such as cough, abdominal pain, febrilosis, tuberculosis, malaria, tonsillitis, paralysis, diarrhoea, jaundice, amoeba and/or arthritis. This finding is in line with that from other studies conducted in Ethiopia [46, 50, 51] in that higher amount of plant species were used to heal human ailments.

The recorded high number of medicinal plants from Fabaceae and Solanaceae (*n* = 11 each) might be related to the higher adaptation potential of the species in these families over a wider range of elevations. Similarly, studies done elsewhere in Ethiopia [5, 16, 24, 46, 48, 49, 52] and in other countries [53, 54] indicated a relatively higher number of remedial plants under family Fabaceae.

The people in Adwa District highly rely on wild sources to obtain medicinal plants, indicating that the habit of cultivating medicinal plants in homegarden is weak. This shows that natural vegetation in Adwa District is highly exposed to overexploitation. Ethnobotanical studies in different parts of Ethiopia [16, 19, 50, 51] showed that most of the remedial plants were gathered from the wild.

Most reported plant species are herbs. This might be due to easy availability to local people and their abundance. Likewise, most ethnobotanical studies in different parts of Ethiopia [4, 5, 16, 17, 19, 24, 45, 55] and other countries in the world [56–58] reported the dominance of herbs for medicine. However, results reported from Babile District in eastern Ethiopia [48] and from Ada’a District [55] showed that shrubs were there the dominant growth form of medicinal plants.

The high use of leaves could be due to better accessibility, ease of preparation and effectiveness due to higher concentration of bioactive compounds in these parts. Harvesting leaves has less effect on plant species survival, whereas harvesting roots adversely affects plant survival [59]. This finding agrees with most medicinal other plant studies in Ethiopia [4, 16, 45, 50] and other countries in the world [60–62]. Nevertheless, results from Ada’a District, western Ethiopia [55] indicated that roots were there the most popular plant part for remedy preparation.

The wide use of fresh plants in traditional remedies could be due to the higher perceived efficacy of fresh plant parts which could be lost upon drying. Contrary to this, dry forms of remedial plant use have low efficacy due to evaporation and deterioration of bioactive components during drying [45]. Ethnobotanical research in Ethiopia and other countries showed a preference for freshly harvested medicinal plants [3, 5, 19, 24, 49, 55, 63, 64].

Higher frequency of crushed plant use might be related to the simple preparation method mostly only involving use of stones that can done by most individuals. Similarly, the findings of Megersa et al. [46] and Belayneh et al. [48] indicated crushing as the most popular method of remedy preparation.

Some plants have diverse uses, and preparation and application methods for different human diseases. For example, the leaf of *Withania somnifera* (L.) Dunal locally named as ‘*Agol*’ is used to treat different human ailments through various methods of preparation and applications. Boiling the leaf of this plant and mixing with leaves of *Eucalyptus globulus* Labill., roots of *Achyranthes aspera* L. and *Cynoglossum lanceolatum* Forssk., and then inhaling the vapour cures eye infections; crushing the leaf/root and pasting this paste on wounds heals the latter in man; cooking the leaf and inhaling the vapour cures febrile illness ‘*Michi*’; crushing its leaves with water and washing the whole body with this watery mix reportedly heals arthritis (‘*Segri*’) (see Additional file 1).

Most medicinal plant preparations are taken externally by applying to the affected skin part. A similar result was reported by Giday et al. [65]. However, studies conducted in Ethiopia [4, 5, 16, 55] and other countries in the world [2, 58, 66] indicated internal application as the main route of administration.

Even though different prescriptions were practised by the herbal practitioners in Adwa District, still the amount prescribed by healers for both children and adults might not conform to the standard prescriptions as in modern medical literature. Many researchers in Ethiopia and other countries also reported the lack of consistent posology in traditional remedy preparation [3, 45].

Our informants mentioned that traditional medicines in Adwa are prepared with different additives and solvents, such as coffee, tea, honey, milk, oil, butter, salt and/or water to dilute the herbal medicine. These ingredients were claimed to be used to reduce side-effect (as an antidote), improve the taste and lower the chances for poisoning. For instance, mixing the crushed leaf of *Heliotropium cinerascens* Steud. ex DC. with butter and pasting this mix on a shaved part of the head cures dandruff and head wounds (see Additional file 1). Similar findings were reported in Ethiopia and other countries [45–47, 49, 51, 67, 68] in that similar solvents and as those reported here are used during the preparation of traditional medicines.

There was no significant knowledge difference (*p* > 0.05) in mean number of medicinal plants mentioned by either male or female informants in Adwa District. This shows knowledge is equally shared by all family members, whereby both females and males are responsible for primary health care within their family. Similarly, studies conducted in Ethiopia [16, 24, 45] showed insignificant medicinal plant knowledge differences (*p* > 0.05) between male and female informants. However, findings by Tefera and Kim [5] and Demie et al. [50] indicated that men had better medicinal plant knowledge in their sample population, which is counterintuitive and not confirmed by studies from elsewhere.

As could be expected, key informants were more knowledgeable than general informants (Table 1). This could be attributed to their years’ long experience and high level of secrecy in using medicinal plants. A similar result was also reported by Giday et al. [65]. However, this finding disagrees with that obtained by Demie et al. [50] who indicated insignificant knowledge differences between key and general informants on medicinal plants used.

The significant difference (*p* < 0.05) observed between age groups shows that the knowledge of youngsters on traditional medicinal plants was low compared to that of adults and elders (Table 1). This is an indication of the decline of traditional remedial plant knowledge in Adwa District. Factors such as modern education, formal health service and the oral transmission might explain this lower knowledge level. In addition, the better knowledge of older people might be the result of their long contact and experience with plants and associated therapeutic uses. The same results were reported in different parts of Ethiopia [5, 16, 24] in that young people have less knowledge compared to their elders.

The observed significant difference in mean number of medicinal plants reported among different education levels (*p* < 0.05) in the study area shows that illiterates and informants who only had minimum formal education were more knowledgeable (Table 1). This indicates that illiterates hold more knowledge than more educated respondents. This might be due to the negative impact of modern education on traditional medicine knowledge. This result agrees with results from a study by Kidane et al. [16] who indicated that illiterate respondents had better knowledge of medicinal plants.

The higher informant consensus factor values as in febrilosis, sudden sickness and headache might be indicative of the presence of similar ethnomedicinal plant knowledge and its continued use in similar ways over different communities [46, 47, 51, 69]. According to Heinrich et al. [42], a high ICF value is vital to discover species with higher probability of containing interesting bioactive components. A high informant consensus factor is also an important argument when it comes to deciding which species to conserve in an environment that is under pressure of having medicinal plant species gradually disappearing. Similarly, the highest value of ICF for febrilosis illness was reported by Kidane et al. [16] from Ganta Afeshum District in Northern Ethiopia.

The highest fidelity level value reported for *Kalanchoe quartiniana* A. Rich. 0.98 (98%) against black leg in livestock could be considered as an indicator for the high healing potential of these plant species (especially against these ailments). Plants with high fidelity levels can be targeted for efficacy investigation of their bioactive ingredients [42, 70]. Similar findings were reported in Ethiopia [16, 45, 49, 68] in that species such as *Rhamnus prinoides* L’Her. and *Withania somnifera* (L.) Dunal were also reported to have very high fidelity levels.

The higher preference ranking of *Cucumis ficifolius* A. Rich. and *Solanum incanum* L. in the treatment of abdominal pains should also lead to further phytochemical analysis, pharmacological investigation and conservation measures. Ethnobotanical investigations done by Teklay et al. [47] confirmed that *C. ficifolius* A. Rich. was used to cure abdominal pains.

*Olea europaea* L. was the most multi-used plant (250) in Adwa District followed by *Cordia africana* Lam. (248). However, multi-use plant species, particularly the top-ranked ones such as *O. europaea* L., will probably be under pressure of overharvesting in the future. Hence, complementary conservation action is urgently needed to protect this multipurpose medicinal plant species from disappearing. Ethnobotanical investigations in Ethiopia [46, 47, 55] also showed that *O. europaea* L. and *Cordia africana* Lam were multipurpose plant species in their respective study areas.

The high popularity of *Kalanchoe quartiniana* A. Rich. is also an argument for further laboratory analysis. However, the popularity of *K. quartiniana* A. Rich. being the most cited and most effective medicinal plant may be indicative of potential danger of overharvesting of this species. Hence, such species should be prioritized for conservation, or even propagation tests as *Kalanchoe* species are often easy-to-propagate.

During group discussions, key informants confirmed that a number of medicinal plant species are indeed disappearing in their environment due to both natural and anthropogenic factors. For instance, most agreed that such species as *Periploca linearifolia* Quart.-Dill. & A. Rich.*, Boscia salicifilia* Oliv., *Cucumis ficifolius* A. Rich. and *Zehneria scabra* Sond. are disappearing from their local area. The highest threat to medicinal plants in Adwa District was confirmed to be agricultural expansion. It should be stressed that plants with multipurpose uses such as *Olea europaea* L. and *Cordia africana* Lam. are most highly affected. Similar results were reported from elsewhere in Ethiopia [5, 17, 46] in that agricultural expansion is the main conservation challenge to medicinal plant survival. Besides, these key informants confirmed that the younger generations are reluctant and even refuse to try and get to know or use traditional medicine. Hence, a lot of invaluable information could be lost whenever traditional medicinal practitioners die without sharing their knowledge with others.

Most informants in Adwa District recommended planting of medicinal plants as the best approach to conserve and protect herbal medicines, followed by soil and water conservation, bringing in protected areas and raising awareness of local people on conservation issues. Homegardens in Adwa District (could) have a vital role in conserving medicinal plant species. The rich diversity of remedial plants in homegarden was obtained by cultivating and protecting a mixture of annual and perennial herbs and woody perennials. Cultivating useful plants in homegardens and protecting and conserving wild natural setting (in situ) is crucial to guarantee future access to herbal medicine for maintaining the primary healthcare system of rural communities but also to be able to provide materials for new chemical lead discovery through laboratory investigation.

## Conclusions

Adwa District is relatively rich in medicinal plant diversity and traditional knowledge about their use, preparation and application, which is still pretty much alive local people. Overall, 127 plant species were documented to treat different ailments. Locally preferred treatments (mentioned by traditional healers) in the area target febrilosis, sudden sickness and spider poison and are preferred over modern treatment. A number of these medicinal plants are under pressure due to overharvesting, but also because of agricultural expansion. Moreover, secrecy, oral-based knowledge transfer and reluctance of young generations to acquire medicinal plant knowledge erode the local indigenous knowledge systems. Therefore, awareness creation should be facilitated to conserve and preserve traditional knowledge but also the medicinal plant species themselves, particularly species that are mainly harvested for their roots, such as *Carissa spinarum* L., *Abutilon mauritianum* (Jacq.) Medik., *Capparis tomentosa* Lam. and *Maytenus arbutifolia* (Hochst. ex A. Rich.) Wilczec. Besides, local people should minimize oral administration to reduce side-effect of unstandardized remedies’ posology. Local communities should be actively involved in conservation and management of remedial plant resources and their indigenous knowledge in their locality. The local people in Adwa District have to be encouraged to cultivate multipurpose plants such as *Olea europaea* L. and *Cordia africana* Lam. There is also a need for development of priority areas for community-managed forest (park) establishment in the district for the conservation of forests in general and medicinal plants in particular.

## Supplementary Information

**Additional file 1: **List of medicinal plants, habits, part used, condition and ways of preparation, route of administration, ailment treated and collection number in Adwa District.

## Data Availability

All data collected for this study were analysed, interpreted and included in this manuscript, and its supplementary materials were attached as Additionafile1.

## Abbreviations

ANOVA: Analysis of variance
CSA: Central Statistical Agency
WHO: World Health Organization
ICF: Informant consensus factor
HH: Household
