# Supplementary material for: Ethnobotanical study of medicinal plants in Adwa District, Central Zone of Tigray Regional State, Northern Ethiopia
Source: J Ethnobiol Ethnomed. 2021 Dec 24;17:71. doi: 10.1186/s13002-021-00498-1 (PMC8709991; doi:10.1186/s13002-021-00498-1)
Supplement: Supplementary file 1 — Additional file 1: List of medicinal plants, habits, part used, condition and ways of preparation, route of administration, ailment treated and collection number in Adwa District. [file 13002_2021_498_MOESM1_ESM.docx]

**Additional file 1:** List of medicinal plants, habits, part used, condition and ways of preparation, route of administration, ailment treated and collection number in Adwa District.

| Scientific  name | Family | Local name | Habit | Part used | Condition of preparation | Way of  preparation | Route of  Administration | Ailment  treated | Other use | Collection number |
| --- | --- | --- | --- | --- | --- | --- | --- | --- | --- | --- |
| *Abutilon bidentatum* Hochst. ex A.Rich. | Malvaceae | Niger  negarit | Shrub | Leaf | Fresh | Leaves are crushed, squeezed and a cup of juice is taken orally | Oral | Stomach ache  ‘*Tsidak*’ |  | LG 011 |
| *Abutilon mauritianum* [(Jacq.)Medik.](https://www.google.com/url?sa=t&rct=j&q=&esrc=s&source=web&cd=14&cad=rja&uact=8&ved=2ahUKEwjnq834w_nhAhXIlosKHY6qDVgQFjANegQIBRAB&url=https%3A%2F%2Fplants.jstor.org%2Fstable%2F10.5555%2Fal.ap.flora.flos000284&usg=AOvVaw0_4AUFcUhuabE6PcbCLL9P) | Malvaceae | Amee | Herb | Root | Fresh | Root is chopped and tied on the waist | Tied | Sprain |  | LG 037 |
| Acacia albida Delile | Fabaceae | Memona | Tree | Hybrid | Dried | Crush and apply on affected part | Dermal | Herpes zoster | Firewood,  fence | LG 031 |
| Acacia etbaica Schweinf. | Fabaceae | Seraw | Tree | Leaf | Fresh | Leaves are roasted on metal plate, powdered and mixed with pure butter and smeared on affected part | Dermal | Dandruff | Fumigation milk, Fire wood, Charcoal | LG 017 |
|  |  |  |  |  |  | Crush the leaves, squeeze the juice, filter and drink it | Oral | Diarrhea  (Goats, Sheep) |  |  |
|  |  |  |  |  |  | Cut the leaves and smeared the latex on the spider poison part | Dermal | wound  spider poison |  |  |
| *Acacia lahai* Benth. | Fabaceae | Lehay | Shrub | Bark | Fresh | Chew the root and hold on affected tooth | Oral | Tooth ache,  Tuberculosis | Fence | LG 035 |
|  |  |  |  |  |  | Crush the fresh bark and soak in water, filter, drink with ‘*Tela*’ (local drink) | Oral | Paralyzes |  |  |
| Achyranthes aspera L. | Amaranthaceae | Mecholo | Herb | Root ,  Whole part | Fresh | Root is chewed and spit on the infected eye | Dermal | Eye ache, (Both) |  | LG 038 |
|  |  |  |  |  |  | Fresh leaf and stem are put on fire and fumigated | Fumigation | Uterus problem |  |  |
| Acokanthera schimperi (A.DC.) Schweinf. | Apocynaceae | Mebtie | Tree | Stem | Fresh | Leaves are crushed and pasted on the affected part | Dermal | Itching/ scabies,  Insecticide |  | LG 073 |
| Adansonia digitata L. | Bombacaceae | Dima | Tree | Bark | Dried | Bark is crushed , mixed with honey and eaten before breakfast | Oral | Impotence |  | LG 007 |
| *Albizi malacophylla* (A.Rich.) Walp. | Fabaceae | Chigono | Tree | Root | Fresh | Fresh root bark is chewed and spit on the infected eye | Inner /Dermal | Eye infection  (livestock) | Fire wood, charcoal | LG 078 |
| *Albuca abyssinica* Jacq*.* | Xygophyllaceae | Shigurtizbei | Herb | Bulb | Fresh | Roots and leaves are crushed and mixed with *Solanum nigrum,* rubbed on the legs | Dermal | Elephantiasis |  | LG 051 |
|  |  |  |  |  |  | Roots and leaves are crushed and mixed with *Boscia angustifolia* and Boscia salicifolia, add wheat flour and paste on the wound | Dermal | Wound  ‘*Lkft qusl*’ |  |  |
| Allium sativum L. | Alliaceae | Tsaeda  shgurti | Herb | Seed | Fresh | Eat or smell the part or crush and eat it with honey | Oral | Cough | Spice | LG 097 |
| Aloe elegans Tod. | Aloaceae | Ere Wedi | Shrub | Latex |  | Roots chopped into seven pieces are put on clean thread and tied on the waist until recovered from the disease | Tied | Sprain  (Both) | Fence | LG 018 |
| *Aloe vera* (L.) Burm.f. | Aloaceae | Ere Anesteyity | Shrub | Latex,  Root |  | Cut a leaf and spread the latex on the wound until healed | Dermal | Any wound, Pimple | Fence | LG 112 |
|  |  |  |  |  |  | Root is chopped in to seven pieces and tied on waist or any part | Tied | Sprain,  (Both) |  |  |
|  |  |  |  |  |  | Coffee cup of exudates is taken every morning for long period of time | Oral | Malaria |  |  |
| Argemone mexicana L. | Papaveraceae | Dandier  netaee | Herb | Latex |  | The wound is smeared by the latex until treated | Dermal | External wound | Seeds used baking *injera* (local food) | LG 033 |
| Artemisia abyssinica Sch.Bip. ex A.Rich. | Asteraceae | Tsitetebayita/ Chenabaryia | Herb | Whole | Fresh, dried | The root is chewed and the juice is swallowed | Oral | Abdominal pain |  | LG 116 |
|  |  |  |  |  |  | Aroma of the leaves help in expelling evil eye | Nasal | Evil eye, |  |  |
| *Azadirachta indica* A.Juss. | Meliaceae | Nim | Tree | Leaf | Fresh | Crush, filter and drink the juice as water  N.B =Little amount for human | Oral | Hen disease’s  and abdominal pain  (human) | Shade, fire wood | LG 006 |
|  |  |  |  |  |  | Crush and squeeze the leaf without water and spray on the location of termites |  | For protection of Termites  ‘Anti -termite ‘ |  |  |
| *Balanites aegyptiacus* (L.) Delile | Xygophyllaceae | Mekie | Tree | Fruit | Fresh | Fruits is directly eaten | Oral | Abdominal parasites | Fence | LG 121 |
| Bidens camporum (Hutch.) Mesfin | Asteraceae | Tselimeteneg | Herb | whole | Fresh | Crush the whole plant, filter with clean cotton, apply three drops of the liquid into the infected eye | Drop | Eye infection | Forage | LG 036 |
| *Boscia angustifolia* A.Rich. | Capparaceae | Kermed | Tree | Leaf | Fresh | The bark is tied with necklace | Tied | Evil sprit |  | LG 034 |
| Boscia salicifolia Oliv. | Capparaceae | Awo | Tree | leaf | Fresh | Fresh leaf is crushed and soaked with water for three days and then the body is washed with it for 5-7 consecutive days or until recovery | Dermal | Paralyzes,  Arthritis |  | LG 074 |
| *Brassica carinata* A.Braun | Brassicaceae | Gurm  ba | Herb | Leaf | Fresh | Leaf is crushed and rubbed on the skin | Dermal | *Quaqusho* |  | LG 019 |
|  |  |  |  |  |  | Chew and swallow the fluid | Oral | Tape worm |  |  |
| *Brassica nigra* (L.) K.Koch | Brassicaceae | Adri | Herb | Fruit , Leaf | Dried | Seed is put on fire and fumigated | Fumigation | Evil eye | Vegetable | LG 098 |
|  |  |  |  |  |  | Leaf is crushed and rubbed on the skin | Dermal | Tinia scaplis  ‘*Quoqusho*’ |  |  |
| *Brucea antidysenterica* J.F.Mill.  . | Simaroubaceae | Malita | Shrub | Leaf ,  Stem and Root  , | Fresh | Boil the leaf in water and wash the body with it | Washing | Epilepsy |  | LG 052 |
|  |  |  |  |  |  | Roasting the stem and burning the swelling part with it | Dermal | Swelling  ‘*Amelo*’ |  |  |
|  |  |  |  |  |  | Root is boiled in milk and drunk | Oral | Rectal prolapsed |  |  |
|  |  |  |  |  |  | The leaves are crushed, salt and butter are added into it and pasted on the wound | Dermal | Wound  ‘*Gizwa*’ |  |  |
|  |  |  |  |  |  | Pound the dry root, mix with roots of Verbascum sinaiticum and *Securidaca longipedunculata* and smear by adding butter on the whole body | Dermal | Jaundice |  |  |
|  |  |  |  |  |  | By surgeon, apices of leaf is inserted into the external skin | Inner | Swelling  ‘*Zigag*’  (Cattle) |  |  |
| Buddleja polystachya Fresen. | Budlejaceae | Metere | Shrub | Root | Fresh | Root is crushed, mixed with *Ruta chalepensis*, *Phoenix reclinata*, *Capparis tomentosa*  and Allium *sativum,* put on fire and fumigated | Fumigation | Evil eye  (Both) |  | LG 124 |
| Calotropis procera (Aiton) Dryand. | Asclepiadaceae | Gindae | Shrub | Latex |  | Milky latex is smeared on the wound for three consecutive days | Dermal | Expel |  | LG 005 |
| *Calpurnia aurea* (Aiton) Benth. | Fabaceae | Hitsawts | Tree | Leaf | Fresh | leaves are crushed and the exudates are applied on the infected skin | Dermal | Mange  (Cattle) |  | LG 111 |
| *Capparis tomentosa* Lam. | Capparaceae | Andyel | Climber | Root | Dried | Root is pounded, mixed with *Phoenix reclinata*, Buddleja polystachya, *Casimiroa edulis* and fumigated on fire | Fumigation | Evil eye,  evil sprit |  | LG 053 |
| *Capsella bursa-pastoris* (L.) Medik. | Brassicaceae | Kimikmo | Herb | Leaf | Fresh | Crush the leaves and paste on the wound | Paste | ‘*qokusho*’ |  | LG 126 |
| *Carica papaya* L. | Caricaceae | Papaya | Shrub | Leaf | Fresh | A coffee cup seeds are chewed and swallowed | Oral | Internal parasites | Edible fruit | LG 075 |
|  |  |  |  |  |  | Leaves are cooked in water, mixed with sugar, and drunk | Oral | Malaria |  |  |
| *Carissa spinarum* L. | Apocynaceae | Agam | Shrub | Root | Both | Dry root is crushed, put on the fire and fumigated nasally | Fumigation | Evil eye | Forage | LG 020 |
| *Casimiroa edulis* La Llave | Rutaceae | Kazmir | Shrub | Leaf | Fresh | Fresh leaf is crushed, mixed with fresh butter and taken orally | Oral | Shivering  ‘*Halafien*’ | Edible fruit | LG 117 |
| *Chenopodium murale* L. | Chenopodiaceae | Hmdmado | Herb | Leaf | Fresh | The leaves are crushed with little amount of water, mixed with fresh butter, and pasted on the wound of the head | Dermal | Abscess and Wound  Trauma Dandruff ' |  | LG 039 |
| *Chenopodium opulifolium* Schrad. ex W.D.J.Koch & Ziz | Chenopodiaceae | Hamliqiboo | Herb | Leaf | Fresh | Crush and place on the wound | Dermal | Wound |  | LG 054 |
| *Cissus petiolata* Hook.f. | Vitaceae | Alke | Climber | Leaf,  Root | Fresh | Chew the root and swallow the juice | Oral | Snake bite  (Human) | Expel snake from  ‘*Tela*’ | LG 099 |
|  |  |  |  |  |  | Stem is tied on the neck of infected livestock | Tied | Snakebite  ‘Livestock’ |  |  |
|  |  |  |  |  |  | Roots are pounded into powder, mixed with root of *C. spinarum* and *C. tomentosa,* put on the fire and fumigated | Fumigation | Evil eye  (Human) |  |  |
| *Citrus limon* (L.) Obseck | Rutaceae | Lomin | Tree | Fruit | Fresh | The fruit juice is squeezed, mixed with Alliums sativa and salt, and rubbed respectively for three consecutive days | Dermal | Hemorrhoids |  | LG 076 |
| *Clematis hirsuta* Guill. & Perr. | Ranunculaceae | Hazobereka | Shrub | Leaf |  | Fresh leaves are crushed and the aroma is smelt | Nasal | Headache |  | LG 021 |
| *Clerodendrum myricoides* (Hochst.) R.Br. ex Vatke | Lamiaceae | Sur-betri | Shrub | Stem | Fresh | Stem is painted with butter, roasted on fire and the swelled part between joints is burned | Dermal | Swelling around joints ‘*Amelo*’ | Make circle  Local  ‘*mekubaeti*’ | LG 004 |
|  |  |  |  |  |  | The stem with latex *of Euphorbia abyssinica* is painted on the swelled part | Dermal | Swelling around neck  ‘*Zigag*’  (Livestock) |  |  |
| *Coffea arabica* L. | Rubiaceae | Buna | tree | Seed | Dried | Powder of roasted seeds are sprayed on the wound until treated | Spray | Wound |  | LG 113 |
| Commicarpus grandiflorus (A. Rich.) Standl. | Asteraceae | Eznitaewa | Herb | Root | Fresh | Fresh root is chewed | Oral | Scorpion bite | Fodder for animals | LG 055 |
| Cordia africana Lam. | Boraginaceae | Awhi | Tree | Leaf | Fresh | Crush, filter and drink it alone or with coffee | Oral | ‘*Mich*’ Acute febrile illness | Eat its Fruit | LG 088 |
|  |  |  |  |  |  | Chew and swallow the fluid in the morning before food | Oral | Abdominal pain ‘*Tsidak*‘ |  |  |
| Croton macrostachyus Hochst. ex Delile | Euphorbiaceae | Tambok | Tree | Leaf | Fresh | Fresh leaf is crushed with water and squeezed, its juice is drunk | Oral | Jaundice | Construction, shade,  For washing pot | LG 048 |
|  |  |  |  |  |  | Cut the apical leaf exudates of the latex and smear on the skin | Dermal | Spider poison  ‘*Tewsas*’ |  |  |
|  |  |  |  |  |  | Seven juvenile leaves are crushed and squeezed, and its juice is drunk | Oral | Yellow fever |  |  |
| *Cucumis ficifolius* A.Rich. | Cucurbitaceae | Leminbaita | Herb | Root | Both | Fruit juice is added into cup of tea and drunk every morning before breakfast | Oral | Blood pressure |  | LG 123 |
|  |  |  |  |  |  | Fruit juice is decocted, sugar is added into it, and taken orally | Oral | Cough |  |  |
|  |  |  |  |  |  | Fresh root is chewed before the meal | Oral | Abdominal pain |  |  |
| *Cucurbita pepo* L. | Cucurbitaceae | Duba | Climber | Seed | Dried | Dry seed is roasted and eaten before meal | Oral | Tape worm, Amoeba | Food  for human | LG 056 |
|  |  |  |  |  |  | Fruits are cooked and taken as soup | Oral | Urine retention |  |  |
| *Cynoglossum lanceolatum* Forssk. | Boraginaceae | Tenegbegiee | Herb | Leaf | Both | Leaves and stem are put on fire and the smoke is inhaled by the patient | Inhalation | Eye disease |  | LG 118 |
| Cyphostemma junceum (Baker) Desc. ex Wild & R.B.Drumm. | Vitaceae | Etsezewie | Herb | Root bark | Both | Finger-sized root is chewed and swallowed  N.B=. Overdose causes severe stomach ache and vomiting | Oral | Snake bite |  | LG 022 |
| *Datura stramonium* L. | Solanaceae | Mezerbae  ashaku | Herb | Fruit | Dried | Seeds are roasted on iron sheet, mixed with butter and the smoke is inhaled by the patient | Fumigation | Tooth ache |  | LG 089 |
| *Dichrostachys cinerea* (L.) Wight & Arn. | Fabaceae | Gonok | Shrub | Stem | fresh | A piece of cloth of the patient is tied on the plant *Dichrosta chyscinerea* | Tied | ‘*Wugat*’ | Firewood | LG 057 |
| *Dodonia angustifolia* L.f. | Sapindaceae | Tahsos | Shrub | Stem  (Hybrid) | Both | Dry or fresh stem piece is tied around fractured leg bone | Tied | Bone fracture  (livestock) | Fire wood, Charcoal | LG 041 |
|  |  |  |  |  |  | The lotion produced during burning of the stem is smeared on wound | Dermal | Spider poison  ‘*Tewsas*’ |  |  |
|  |  |  |  |  |  | Hybrid stem protect from Gun fire | Tied | Gunfire |  |  |
| *Echinops kebericho* Mesfin | Asteraceae | Dandier  ashak | Herb | Stem | Fresh | The stem is roasted and the swelled part is burned with it | Dermal | Wart  ‘*Tibuadgi* ‘ |  | LG 002 |
| *Ehretia cymosa* Thonn. | Boraginaceae | Kirwah | Tree | Stem | Fresh | Stem is roasted on fire and the stroma (swelled) part of body is burned with it | Burn | Swell  ‘*Amelo*’ | Farming  materials | LG 086 |
| Eucalyptus camaldulensis Dehnh. | Myrtaceae | Keyhbahrizaf | Tree | Leaf | Fresh | The leaf is boiled with water and its vapour is inhaled and fumigated | Fumigation | Eye infection  and Cough | Construction, fire wood | LG 025 |
| Eucalyptus *globulus* Labill. | Myrtaceae | Tsaeda  bahrizaf | Tree | Leaf | Fresh | During the period delivery, woman take its leaf through reproductive part | Sit | Protect  bleeding | Fire wood,  Construction | LG 100 |
|  |  |  |  |  |  | Leaves are crushed and smeared on affected part | Dermal | Anthrax ,  Wound |  |  |
|  |  |  |  |  |  | Boil the leaf by mixing with leaves of and Withania *somnifera* in water and inhale its vapour | Inhalation | Common cold,  Cough and Eye infection /*Gerefta*/ |  |  |
| Euclea *racemosa* L. | Ebenaceae | Kulio | Shrub | Root ,leaf | Fresh | Fresh leaf is crushed and placed on the bleeding part | Dermal | Bleeding injury | Fire wood | LG 059 |
| *Euphorbia abyssinica* J.F.Gmel. | Euphorbiaceae | Kolqol | Tree | Latex |  | Smear the swelling part with the exudates of the latex | Dermal | Swelling  ‘Zigaz’ | Fire wood | LG 023 |
|  |  |  |  |  |  | Apply the latex on the swelled part | Dermal | Tuberculosis |  |  |
| Euphorbia petitiana A.Rich. | Euphorbeiaceae | Tsebadimu | Herb | Latex |  | Fresh leaf is crushed and rubbed on the skin | Dermal | Teeth pain |  | LG 110 |
|  |  |  |  |  |  | Latex is spread on bread and eaten | Oral | Abdominal pain |  |  |
| *Euphorbia tirucalli* L. | Euphorbiaceae | Kinchib | Shrub | Latex |  | Burn the swelled part by roasting the steam on fire | Dermal | ‘*Amelo*’ | Fence | LG 079 |
| *Ficus palmate* Forssk. | Moraceae | Beles | Tree | Stem , latex |  | The wound is smeared by the milky latex until treated | Dermal | External  Wound |  | LG 042 |
|  |  |  |  |  |  | Stem is roasted on fire and the swelled part of body is burned by it | Dermal | Wart  ‘*Tibuadgi*’ |  |  |
| Ficus vasta Forssk. | Moraceae | Daero | Tree | Diqala | Dried | A pieces of *diqal*a (epiphyte) is inserted into milk and drunk | Fumigate | Milk | Shade | LG 119 |
|  |  |  |  |  |  | Stem of epiphyte is roasted on fire and the swelled part of the body is burned with it | Burned | ‘*Amelo*’ |  |  |
| *Foeniculum vulgare* Mill. | Apiaceae | Shilan | Herb | leaf |  | Take a solution of the plant boiled in water or with tea | Oral | Urine retention | For tea | LG 024 |
| *Grewia ferruginea* Hochst. ex A.Rich. | Malvaceae | Tsinquya | Tree | Leaf |  | Fresh leaves and stem are crushed with water and its juice is taken orally | Oral | Retained fatal placenta | Fire wood, | LG 125 |
| Guizotia abyssinica (L.f.) Cass. | Asteraceae | Noug | Herb | Fruit | Dried | The seeds are pounded, boiled and drunk with a broth every night before sleeping | Oral | Intestinal parasite | Food | LG 087 |
| *Heliotropium cinerascens* Steud. ex DC. | Boraginaceae | Amamgmel | Herb | Leaf | Fresh | Crushing and mixing with butter, and pasting on shaved part of head | Dermal | Dandruff,  wound of head |  | LG 001 |
| *Heteromorpha arborescens* (Spreng.) Cham. & Schltdl. | Apiaceae | Mrkuzzbei | Shrub | Leaf | Fresh | Leaves are put on fire and fumigated | Fumigation | ‘Mich ‘ |  | LG 080 |
| Hibiscus micranthus L.f. | Malvaceae | Sgothamat | Herb | Root,  Leaf | Fresh | Chew and swallow the fluid | Oral | Abdominal pain  ‘*Tsidaq*’  Abdominal pain | ‘*Shig* for *Hoya*  *hoye*’ | LG 047 |
| *Hordeum vulgare* L. | Poaceae | Sigem | Herb | Seed | Fresh | Fresh leaf is crushed and rubbed on the skin | Dermal | ‘*Qaqusho*’ | Cereal | LG 091 |
| *Huernia macrocarpa* Schweinf. ex K.Schum. | Asclepiadaceae | Ango | Herb | Leaf | Fresh | Leaf is burned on fire and applied on the affected part | Dermal | Hemorrhoids |  | LG 026 |
| *Impatiens tinctoria* A.Rich | Balsaminaceae | Enshoshela | Shrub | Tuber | Fresh | The tuber is crushed, boiled, squeezed then drunk with coffee cup | Oral | Arthritis |  | LG 016 |
| *Indigofera arrecta* A.Rich. | Fabaceae | Enkoegri | Shrub | Leaf | Fresh | The leaf is crushed and evolved by leaf of Cordia africana and then eaten | Oral | Acute swelling  (*Megerem*) |  | LG 122 |
| Jasminum abyssinicum Hochst. ex DC. | Oleaceae | Habitselim | Climber | Leaf | Fresh | Leaves are crushed, squeezed and cup of juice with sugar is taken orally | Oral | Abdominal parasites |  | LG 058 |
| *Justicia schimperiana* (Hochst ex. Nees) T. Andres | Acanthaceae | Simza | Shrub | Leaf | Fresh | Fresh leaf is crushed and mixed with fresh butter, rubbed on the skin at day time and wash at night | Dermal | Itching,  herpes |  | LG 114 |
|  |  |  |  |  |  | Leaves are crushed and squeezed, and the juice is drunk | Oral | Diarrhea  (livestock) |  |  |
|  |  |  |  |  |  | The leaf mixed with leaves of Eucalyptus globulus, Zehneria scabra is boiled with water, fumigated once every day for three consecutive days | Fumigate | Febrile illness ‘*Mich*’ |  |  |
| *Kalanchoe quartiniana* A.Rich | Crassulaceae | Adaequa | Herb | All part | Fresh | All parts are crushed, the water is added and mixed with butter and taken orally | Oral | Black leg  ‘*Halafie*‘  (Cattle) | Protect  Termites | LG 043 |
| *Lasiocarphus ferrugineus* Pohl ex Baker | Fabaceae | Hambohambobayta | Herb | Leaf |  | Boil it in water by mixing with leaves of E. globulus and inhale the vapour | Inhale | Cough |  | LG 032 |
|  |  |  |  |  |  | Boil it in water and wash the whole body for three days | Washing | ‘*Mitat* ‘ |  |  |
|  |  |  |  |  |  | Crush, filter and drink | Oral | Anti-bleeding |  |  |
| Leonotis nepetifolia (L.) R.Br. | Lamiaceae | Teketater | Herb | Whole | Both | Fumigating oneself with smoke of plant | Fumigation | Eye ache  ‘*michi*’ |  | LG 090 |
| Lepidium sativum L. | Brassicaceae | Shinfae | Herb | Seed | Dried | The seed is crushed, covered with piece of clothes, and tied on neck and smelt | Oral | Diarrhea ‘  Injury, bleeding | Expel evil eye | LG 060 |
|  |  |  |  |  |  | The seeds are ground, powdered and added into water and the solution is sprayed at the indoor | Dermal | Expel evil sprit  (Both) |  |  |
| Linum usitatissimum L. | Linaceae | Entatie | Herb | Fruit | Dried | The seed is Pounded, mixed with water, and drunk | Oral | Constipation | Soup, food | LG 003 |
|  |  |  |  |  |  | The seed is decocted, mixed with sugar, and drunk | Oral | Retained fatal placenta |  |  |
| *Lippia javanica* (Burm.f.) Spreng. | Verbenaceae | Ksihe | Herb | Leaf | Fresh | The backbone of the equine is rubbed by the fresh leaf | Hit | Back bone fracture  Equine  ‘*Abriq*’ | Marvelous odor, as mat | LG 085 |
| Lycopersicon esculentum Mill | Solanaceae | Tsebhiabun | Herb | Leaf | Fresh | Fresh leaves are smashed and mixed with water then one cup of tea is taken | Nasal | To expel leech, ‘*Haseka resi’* |  | LG 077 |
|  |  |  |  |  |  | Leaves are crushed, mixed with honey and pasted on the wound | Dermal | Wound  ‘*Timto*’ | It use  For sauce, food |  |
| *Maesa lanceolata* Forssk. | Primulaceae | Kelewa | Shrub | Root | Fresh | Boiled and the decoction is drunk when cooled | Oral | Constipation |  | LG 012 |
| Maytenus arbutifolia (Hochst. ex A.Rich.) Wilczek | Celastraceae | Atat | Shrub | Root | Dried | Root is pounded, powdered, mixed with root of *C. spinarum*, put on the fire and fumigated | Fumigation | Evil eye | Fire wood | LG 067 |
| Melia azadirachta L. | Meliaceae | Nim Areb | Tree | Leaf | Fresh | Leaf is crushed, squeezed using gauze or clean cloth and its juice is drunk | Oral | Intestinal parasite (human) | Shade, fire wood | LG 069 |
|  |  |  |  |  |  | Fresh leaves are crushed with small amount of water and washing the skin with it | Dermal | Etoparasite like lice (Livestock) |  |  |
| *Nicandra physalodes* (L.) Gaertn. | Solanaceae | Mezerbae/absho | Herb | Leaf | Fresh | Fresh leaf is crushed and applied topically | Dermal | Ecto-parasite  (Livestock) |  | LG 040 |
|  |  |  |  |  |  | Leaves are crushed, squeezed, filtered and a cup of juice is taken for three days | Oral | Brain sharpness |  |  |
| *Nicotiana glauca* Graham | Solanaceae | Chenawiqotsili | Shrub | Leaf | Fresh | Crush by adding water and smear on affected part or wash with it | Dermal | Ecto-parasite  (livestock) |  | LG 083 |
| *Nicotiana tabacum* L. | Solanaceae | Timbaho | Herb | Leaf | Fresh | Crush and squeeze the leaf, and apply into the nose | Nasal | To expel leech |  | LG 061 |
| *Nigella sativa* L. | Ranunculaceae | Awasda | Herb | Seed | Dried | Seeds are pounded into powder, mixed with little boiled water, and drunk | Oral | Stomach pain | Spice | LG 105 |
| Ocimum lamiifolium Hochst. ex Benth. | Lamiaceae | Demakasie | Herb | Leaf | Fresh | Leaf is crushed and taken with coffee | Oral | ‘*Mich*’ |  | LG 008 |
| Olea europaea L. | Oleaceae | Awlie | Tree | Leaf | Fresh | The leaves are chewed during teeth pain | Oral | Tooth pain | Construction, firewood,  charcoal, fodder, tooth brush | LG 101 |
| Otostegia fruticosa (Forssk.) Schweinf. ex Penzig | Lamiaceae | Sasa | Shrub | Leaf | Fresh | Crush the leaves with little amount of water and filter with clean cloth and drop the infusions in nostrils | Smell /nasal | Tonsillitis |  | LG 062 |
|  |  |  |  |  |  | Crush the leaves with little amount of water and wash the infected skin | Dermal | Ecto-parasite’s  (livestock) |  |  |
| Otostegia integrifolia Benth*.* | Lamiaceae | Chiendog | Shrub | Leaf | Fresh | Put the leaf on fire and fumigate the house | Fumigation | Insecticide Livestock’s |  | LG 109 |
|  |  |  |  |  |  | Chew seven juvenile leaves and swallow the juice | Oral | Abdominal pain |  |  |
| *Periploca linearifolia* Quart. Dill. & A.Rich. | Asclepiadaceae | Moder | Shrub | Leaf,  Stem | Fresh | Leaf and stem are soaked for 1-3 days and the body is washed with it for the 1-7 days | Dermal, | Paralyze  ‘Gusay’ |  | LG 027 |
| *Phoenix reclinata* Jacq. | Arecaceae | Sye | Shrub | Root | Dried | Roots are pounded into powder, mixed with *C. tomentosa*,  Buddleja polystachya, *Casimiroa edulis* and fumigated on fire | Fumigation | Evil eye |  | LG 115 |
| *Phytolacca dodecandra* L’Hér.  . | Phytojaccaceae | Shibti | Shrub | Root ,  Seed | Both | Crush the root, squeeze it, add into it a decanted *Tella* (local drink) and drink the mixture before breakfast | Oral | Rabbis  (Both) | For washing clothes | LG 081 |
|  |  |  |  |  |  | Small piece of root is chewed before breakfast, after a half hour 1L milk is drunk and continued by half hour interval for 24 hours for seven days | Oral | Jaundice |  |  |
|  |  |  |  |  |  | The crushed seeds are homogenized in water and dropped in to eyes | Dermal | Eye infection  (Both) |  |  |
|  |  |  |  |  |  | The crushed root of unfertile *Phytolacca dodecandra* is eaten | Oral | Anthrax (*nefri*)  (Livestock) |  |  |
|  |  |  |  |  |  | Crush the leaves, mix with water and wash the infected skin with it | Dermal | Ecto-parasite |  |  |
|  |  |  |  |  |  | Seeds/fruits is pounded and the bite part (wound) by dog is washed with it | Washing | Rabbis |  |  |
| *Plectranthus ornatus* Codd. | Lamiaceae | Endifdif | Herb | leaf | Dried | fumigating women after delivery and protecting from bad smell | Fumigation | Expel evil eye |  | LG 009 |
| Plumbago zeylanica L. | Plumbaginaceae | Aftuh | Herb | Root  Leaf | Fresh | Soak the root in water by mixing with leaves of R. nervous and whole part of Withania somnifera and wash body with it | Washing | Jaundice |  | LG 120 |
|  |  |  |  |  |  | Leaves are boiled with leaves of Withania somnifera and the whole body is washed with it | Washing | Skin itching, joint pain |  |  |
|  |  |  |  |  |  | Chew the root and spit to the mouth of child | Oral | Tonsil |  |  |
|  |  |  |  |  |  | Chew root and hold in tooth and use as tooth brush | Oral | Tuberculosis |  |  |
|  |  |  |  |  |  | The root is digested by mouth and its piece is inserted into the dissected skin of the animals | Inner | Swelling  ‘*Zigag*’  (cattle) |  |  |
| Rhamnus prinoides L’Her. | Rhamnaceae | Gesho | Shrub | Leaf | Fresh | Juvenile leaf is chewed and spit in to the mouth of paint child | Oral | Tonsil,  Uvula enlargement | Local beverage  (*Tela*) | LG 068 |
| *Rhus glutinosa* Hochst. ex A.Rich. | Anacardiaceae | Mengi | Shrub | Stem  ‘Hybrid’ | Fresh | Stem is roasted on the fire, butter is rubbed added, and the swelled part is burned with it | Burn | Swelling  ‘*Amelo*’ | For bread baker | LG 063 |
| *Rhus natalensis* Bernh. ex C.Krauss | Anacardiaceae | Tetialo | Shrub | Leaf | Fresh | Leaves are crushed, squeezed, and a cup of its solution is taken orally for five consecutive days | Oral | Jaundice | For bread baking | LG 106 |
| *Rosa abyssinica* R.Br. | Rosaceae | Gaqa | Herb | Stem | Both | Seven chopped stems are tied around the neck with necklace | Dermal | Sever wound on head  ‘*Gaqa*’ | Fence | LG 070 |
| Rumex abyssinicus Jacq. | Polygonaceae | Mokmoko | Herb | Root | Dried | Dry root is chopped in to pieces, pounded and drunk with tea, coffee | Oral | Tonsil, Tuberculosis, Tooth ache | For tea spice | LG 013 |
| *Rumex nepalensis* Spreng. | [Polygonaceae](https://en.wikipedia.org/wiki/Polygonaceae) | Shombobaet | Herb | Leaf | Fresh | The leaves are crushed, mixed with Kalanchoe quartiniana, the juice is squeezed and drunk | Oral | Black leg ,  Shivering | Fodder | LG 092 |
| Rumex nervosus Vahl | Polygonaceae | Hakot | Shrub | Leaf |  | Fresh leaf is crushed and rubbed on the skin or leave soaked for one day is spread on affected skin | Dermal | Itchy,  Skin rash  (Human) | For washing ‘*Tela*’ pot | LG 102 |
|  |  |  |  |  |  | Fresh leaves are mixed with leaves of K. quartiniana, C. macrostachyus and R. nepalensis together with water and the juice is squeezed and taken orally | Oral | Black leg,  Shivering  ‘Halafen’ |  |  |
| Ruta chalepensis L. | Rutaceae | Chena  adam | Herb | Leaf | Fresh | Leaves are crushed and smelt | Smell | Evil sprit | Used as spices such as *shro*, milk | LG 044 |
|  |  |  |  |  |  | Fresh leaves are added to tea, coffee or milk and then the adult drink until treated | Oral | Abdominal pain |  |  |
|  |  |  |  |  |  | Leaves are crushed with *Allium sativum*, enclosed by plastic materials or cloth and tied on the neck region of a young to smell it | Tied | Evil spirit,  cough |  |  |
| *Sansevieria erythraeae* Mattei | Dracenaceae | Tirmieqa | Shrub | Leaf | Fresh | Leaves are heated on fire, the juice is squeezed into tea cup and three to four drops are added into the infected ear | Inner | Earache |  | LG 093 |
| *Schinus molle* L*.* | Anacardiaceae | Tikurberbere | Tree | Leaf | Fresh | Crush the leaves evolve by a piece of cloth and smell and paste on the front skull mixed with faeces of birds | Smelled | Tonsillitis, fever | Construction , firewood | LG 108 |
|  |  |  |  |  |  | Leaves are crushed with water and mixed with Rumex nepalensis, Ruta chalepensis and butter, drunk with cup of glass | Oral | Black leg  (cattle) |  |  |
|  |  |  |  |  |  | The leaf is crushed, mixed with Melia Azadirachta, filtered by clean clothes, and the infusions dropped in nostrils | Nasal | ‘Hasekare’,  Leech  (livestock) |  |  |
|  |  |  |  |  |  | Hybrid of *Schinus molle* is tied on the neck | Tied | Repel evil sprit |  |  |
| *Securidaca longipedunculata* Fresen. | Polygalaceae | Shitora | Shrub | Whole | Both | The whole part is chopped, dried and put on fire and fumigated | Fumigate | Evil eye  abdominal problems  (Both) |  | LG 010 |
| *Senecio anteuphorbium*  (L.) Sch.Bip. | Asteraceae | Bierir | Herb | Whole part | Both | Whole part is put on fire and fumigated | Fumigation | Evil eye  ‘*Tselaeisenay*’  (Both) |  | LG 104 |
| *Senna singueana* (Delile) Lock | Fabaceae | Hambohambo | Shrub | Bark , | Fresh | Fresh root bark is chewed | Oral | abdominal pain | Fire wood | LG 049 |
| *Sida ovata* Forssk. | Malvaceae | Dekidaero | Herb | Leaf | fresh | Leaf is crushed, burned and applied on the wound | Dermal | Inflammatory wounds |  | LG 072 |
| Sida schimperiana Hochst. ex A.Rich. | Malvaceae | Tifrarya | Shrub | Root | Fresh | Root is chewed and its juice is drunk | Oral | Abdominal pain | Washing pot of local beer  (*Tela*), tooth brush | LG 084 |
| *Sideroxylon mascatense* (A.DC.) T.D.Penn. | Sapotaceae | Sereret | Tree | Stem,  leaf | Both | Stem and leaf is put on fire and fumigated | Fumigation | Evil eye  ‘Spiritual diseases’  (Both) | For making  Babble cross | LG 050 |
| Silene macrosolen Steud. ex A.Rich. | Caryophyllaceae | Saerisaero | Herb | Whole | Both | The whole part is put on fire and fumigated | Fumigation | Evil eye  (Both) | Expel snake | LG 096 |
| Solanum hirtulum Steud. ex A.Rich. | Solanaceae | Alalimo | Shrub | Leaf | Dried | Dry leaf mixed with butter is smeared on the shaved head of the child | Dermal | Wound on head  ‘*Gaqa*’ | Fence | LG 014 |
| Solanum incanum L. | Solanaceae | Nieshtoyengule | Shrub | Root ,leaf | Fresh | Crushed leaf with honey paste on swelled part and small eaten | Dermal | Acute Swelling | Leather making | LG 107 |
| Solanum marginatum L.f. | Solanaceae | Abiyengule | Shrub | Root,  leaf | Fresh | Root is chopped in to seven pieces and tied on affected part | Tied | Sprain, | Hair remover for skin | LG 046 |
| *Solanum nigrum* L. | Solanaceae | Alem | Herb | Leaf | Fresh | Fresh leaf is crushed and applied topically on the wound | Dermal | Wound | Its fruit is eaten | LG 065 |
| Sorghum bicolor (L.) Moench. | Poaceae | *Liequa* | Herb | Seed | Dried | The root is crushed and pasted on the wound | Dermal | ‘*Almaz balechira*’ | Cereals | LG 103 |
| *Stereospermum kunthianum* Cham. | Bignoniaceae | Argzana | Tree | Leaf,  bark | Fresh | Fresh leaf is crushed, mixed with water and drunk | Oral | Hen’s disease | firewood | LG 028 |
|  |  |  |  |  |  | Fresh stem bark is crushed and applied on wound | Dermal | Skull injury  (head bone) |  |  |
| *Synadenium grantii* Hook.f. | Euphorbiaceae | Tsaedakinchib | Shrub | Leaf | Fresh | Crush leaves and squeeze juice, filter smeared the skin | Dermal | Washing | Fence | LG 094 |
| *Tagetes minuta* L. | Asteraceae | Echefarus | Herb | Leaf | Fresh | Fresh leaf is smelt | Smell | Evil sprit  Evil eye | Forage | LG 071 |
| *Terminalia brownii* Fresen. | Combretaceae | Weyba | Tree | Bark | Fresh | Leaves are crushed, squeezed and filtered with clean cotton and drunk | Oral | Jaundice | Fire wood | LG 015 |
| Trigonella foenum-graecum L | Fabaceae | Abaeke | Herb | Seed | Dried | Seeds are roasted on metal plate, boiled in water and drunk as tea | Oral | Abdominal pain | For milk  Spice  Food | LG 095 |
| Verbascum sinaiticum Benth. | Scrophulariaceae | Tirnaka  /demeketir | Herb | Leaf  Root | Both | Dry leaves are pounded and sprayed on the wound | Dermal | ,  Fire burn wound |  | LG 127 |
| *Verbena officinalis* L. | ‎Verbenaceae | Sriftiet /achit | Herb | whole | Fresh | Crush the leaf and sniff its aroma | Nasal | Respiratory and throat infections |  | LG 082 |
| *Vernonia amygdalina* Del. | Asteraceae | Grar /Grawa | Shrub | Leaf | Fresh | Leaf is boiled with water and the steam is fumigated | Fumigation | ‘*Mich*’  ‘*Gonfi* ‘ |  | LG 029 |
| *Vicia faba* L. | Fabaceae | Aterbahiri | Herb | Seed | Dried | The matured seed is pasted on the pus wound | Dermal | Local swelling and wound | Food  Sauce | LG 066 |
| Withania somnifera (L.) Dunal | Solanaceae | Agol | Herb | Leaf | Fresh | Boil it in water by mixing with leaves of E. globulus, roots of A. aspera and C. lanceolatum and inhale the vapour | Fumigation | Eye infection | Food | LG 045 |
|  |  |  |  |  |  | Leaf /root are crushed and paste on the wound | Dermal | Topical  Wound |  |  |
|  |  |  |  |  |  | Leaves are cooked and the vapour is inhaled to get relief from ‘*Mic*h (Febrile illness). | In hale | ‘*Mich*‘(Febrile illness). |  |  |
|  |  |  |  |  |  | The leaves are crushed and homogenized in water and the whole body is washed with the residue soak | Washing | Arthritis  ‘*Segri*’  Spiritual  disease, |  |  |
| Zehneria scabra Sond. | Cucurbitaceae | Hafaflo | Climber | All part | Fresh | Crush with little amount of water, filter and drink | Oral | ‘*Mich* ‘ |  | LG 064 |
|  |  |  |  |  |  | Leaves are crushed, squeezed, mixed with little water and one beaker is drunk before food | Oral | Abdominal pain, burn sensation |  |  |
| Zingiber officinale Roscoe | Zingiberaceae | Zingible | Herb | Root | Both | Chew the root and swallow the fluid/juice | Oral | Abdominal pain | Spices | LG 030 |
